# Association of Serum Fatty Acids at Admission with the Age of Onset of Intracerebral Hemorrhage

**DOI:** 10.3390/nu12102903

**Published:** 2020-09-23

**Authors:** Takahisa Mori, Kazuhiro Yoshioka, Yuhei Tanno, Shigen Kasakura

**Affiliations:** Department of Stroke Treatment, Shonan Kamakura General Hospital, Okamoto 1370-1, Kamakura City, Kanagawa 247-8533, Japan; y.kazuhiro12@icloud.com (K.Y.); yxip01@icloud.com (Y.T.); kasakura@med.kitasato-u.ac.jp (S.K.)

**Keywords:** serum fatty acids, admission, acute ischemic stroke, age of onset, dihomo-gamma-linolenic acid, docosahexaenoic acid

## Abstract

Dietary triglycerides influence the serum concentrations of fatty acids (FA) and their weight percentages (wt%), which might be associated with the age of onset of intracerebral hemorrhage (ICH). We investigated the correlation between serum FA levels and proportions at admission, and the age at onset of ICH. We included patients admitted between 2016 and 2019 within 24 h of the onset of ICH, and calculated the correlation coefficients between their age, serum FA concentration, and FA wt%. We performed multiple linear regression analysis to identify individual FAs related to the age at onset of ICH. Furthermore, we estimated the threshold values of FAs that were independently associated with the age at onset of ICH <65 years, using receiver operating characteristic curves by logistic regression. Our inclusion criteria were met by 141 patients (mean age, 67 years). The concentration of dihomo-gamma-linolenic acid (DGLA) and the wt% of eicosapentaenoic acid (EPA) were significant independent variables for the age at onset of ICH. The ROC curves for the age of onset <65 years were ≥108.6 µmol/L for DGLA and ≤1.7% for EPA. Increased DGLA concentration and decreased EPA wt% were significantly associated with young-onset ICH.

## 1. Introduction

Triglycerides (TGs) consist of glycerol and three fatty acids (FAs). Elevated levels of serum TGs are a risk factor for vascular diseases [1]. Dietary TGs are derived from meat, fish, and vegetables, and influence the serum TG and FA levels [2]. Hypercholesterolemia is also a risk factor for coronary heart disease [3]. However, low levels of low-density lipoprotein cholesterol (LDL-C) and TGs are risk factors for intracerebral hemorrhage (ICH) according to the Atherosclerosis Risk in Communities Study [4]. Higher consumption of fish and n-3 polyunsaturated fatty acids (n-3 PUFAs) is associated with a reduced risk of thrombotic infarction [5] and coronary artery disease (CAD) [6] in healthy middle-aged subjects; however, it is not related to the risk of hemorrhagic stroke [5]. Patients with onset of acute ischemic stroke at a young age were reported to have high levels of dihomo-gamma-linolenic acid (DGLA) and low levels of docosahexaenoic acid (DHA) wt% at admission [7]. It has been previously reported that intake of saturated fatty acids (SFAs) is inversely associated with the risk of intracerebral hemorrhage in the Japanese population [8,9]. However, serum FA levels were not examined at the onset of the cardiovascular event [5,6,8]. Intake of n-3 PUFAs was inversely associated with the risk of stroke, whereas intake of SFAs or n-6 PUFAs was not associated with the risk of any type of stroke in the Swedish mammography cohort [10]. Few studies have reported an association between serum n-3 PUFA levels and intracerebral hemorrhage (ICH).

The weight percentages (wt%) of individual FAs in the total serum FAs have been examined in healthy humans. With increasing age, wt% of n-6 polyunsaturated fatty acids (n-6 PUFAs) and n-3 polyunsaturated fatty acids (n-3 PUFAs) decreases and increases, respectively [11]. CAD results in increased serum concentrations of palmitic acid (PA), stearic acid (StA), oleic acid (OlA), linoleic acid (LiA), and arachidonic acid (AA), and a lower serum concentration of eicosapentaenoic acid (EPA), compared to the respective concentrations in subjects without CAD [12]. In addition, the wt% values of EPA and DHA were lower in subjects with CAD than in healthy controls [12]. The Japan Public Health Center study reports that the intake of SFAs is inversely associated with lacunar infarction [8]. However, in patients who experienced lacunar or atherosclerotic stroke in their 50s, the serum concentrations of SFAs, n-9 monounsaturated fatty acids (n-9 MUFAs), and n-6 PUFAs were reported to be elevated [13]. It is, therefore, apparent that assessment of serum FA levels may provide different clinical information to questionnaire results on FA intake.

The serum concentrations and wt% of FAs may be related to ICH. Serum levels of lipids and FAs at admission are examined under non-fasting conditions, which are associated with the dietary intake of TGs. In this study, we investigated the association between the serum concentrations and proportions of FAs at admission in patients with ICH, and the age of onset of ICH.

## 2. Materials and Methods 

We conducted a cross-sectional study. The inclusion criteria were patients with ICH who were admitted to our institution between August 2016 and July 2019, within 24 h of the onset of ICH and underwent evaluation of blood lipids and FAs at admission. We excluded patients with a hospital modified Rankin scale score ≥3 before hospitalization, or those with a body mass index <18.5, who were defined as having a severe disability or were underweight respectively, according to the World Health Organization guidelines. These criteria ensured the exclusion of patients with possible malnutrition. 

### 2.1. Measurement of Serum Lipids and Fatty Acids

Serum total cholesterol (T-CHO), TGs, high-density lipoprotein-cholesterol (HDL-C), and LDL-C were measured enzymatically using reagents manufactured by Denka Seiken (Denka Co. Ltd., Chuo, Tokyo, Japan) on a BioMajesty 6050 High Throughput Clinical Chemistry Analyzer (JEOL Ltd., Akishima, Tokyo, Japan). We examined the SFAs, lauric acid (LaA; C12:0), myristic acid (MyA; C14:0), PA (C16:0), and StA (C18:0); the n-9 MUFA OlA (C18:1); the n-6 PUFAs, LiA (C18:2), DGLA (C20:3), and AA (C20:4); and the n-3 PUFAs, alpha-linolenic acid (AlA; C18:3), EPA (C20:5), and DHA (C22:6). We measured the serum concentrations and wt% of each FA at admission. The FAs in 1 mL of serum were measured at BML, Inc. (Shibuya, Tokyo, Japan). FAs were extracted according to the technique described by Bligh and Dyer [14], using tricosanoic acid (Nu-Chek Prep, Inc., Elysian, MN, USA) as an internal standard. Lipid extracts were hydrolyzed, extracted with chloroform, and dried under nitrogen gas. After adding a 30% potassium methoxide methanol solution (FUJIFILM Wako Pure Chemical Corporation, Osaka, Osaka, Japan) to the residual sample, it was incubated at 100 °C for 5 min and then cooled. Samples were extracted with hexane and analyzed on a GC-2010 Plus Capillary Gas Chromatograph (SHIMADZU Corporation, Kyoto, Japan) equipped with a flame ionization detector, using a BPX70 column (30 m × 0.22 mm I.D., 0.25-μm film thickness; SHIMADZU GLC Ltd., Tokyo, Japan). The operating conditions were as follows: 50 °C for 0.5 min, followed by an increase in temperature to 260 °C over 25 min, which was subsequently maintained for 5 min. The injector and detector temperatures were set at 240 °C and 280 °C, respectively, and helium was used as the carrier gas at a flow rate of 1.09 mL/min. Component identification was performed by comparing retention times with those of the respective standards (Sigma-Aldrich Japan, Inc., Meguro, Tokyo, Japan; Nu-Chek Prep, Inc., Elysian, MN, USA). The concentrations of serum lipids and FAs and the wt% of serum FAs were determined using internal standard ratios.

### 2.2. Evaluation

We evaluated patient characteristics, serum levels of lipids (T-CHO, LDL-C, HDL-C, and TG), serum levels of FAs, and their proportions, and identified independent FAs for the age of onset of ICH by multivariate analysis. Most of the fatty acids were present as components of triglycerides or phospholipids, and there was very little free fatty acid. In this study, we wanted to know the concentrations and wt% of individual fatty acids. Therefore, we did not distinguish fatty acids from free fatty acids, triglycerides, and phospholipids.

### 2.3. Ethical Approval

All procedures were performed in accordance with the ethical standards of the institution (Shonan Kamakura General Hospital, Kamakura, Japan) and the 1964 Helsinki Declaration. The Tokusyukai Group Ethical Committee approved this retrospective study (TGE01486-024). 

### 2.4. Consent to Participate 

Written informed consent for participation and publication was not required. The study was based on an opt-out model of enrollment, which was approved by the ethical committee.

### 2.5. Statistical Analysis 

We expressed normally distributed continuous variables as means ± standard deviation and non-normally distributed continuous variables as medians and interquartile ranges. We used a multiple comparison test to compare all possible FA pairs and the Spearman rank correlation coefficient (*r*_s_) to measure the strength of the relationships between variables. We defined 0 ≤ |*r*_s_| < 0.1 as no correlation, 0.1 ≤ |*r*_s_| < 0.4 as a weak correlation, 0.4 ≤ |*r*_s_| < 0.6 as a moderate correlation, and 0.6 ≤ |*r*_s_| as a strong correlation. We defined multicollinearity as a strong correlation between the variables. After excluding FAs with multicollinearity, we performed multiple linear regression analyses to identify FAs independently affecting the age at onset of ICH. We used the Durbin–Watson (D–W) ratio for the residual analysis of multiple linear regression, and a D–W ratio > 1.5 and <2.5 was considered as appropriate. Multiple logistic regression analysis was also used to find independent FAs that correlated with the age at onset of ICH < 50 years, <65 years, and <80 years. We estimated the threshold values of FAs for the onset of ICH at age < 50 years, <65 years, or <70 years using area under the curve (AUC) values derived from the receiver operating characteristic (ROC) curves of the logistic regression model. A value of *p* < 0.05 was considered statistically significant. For all statistical analyses, we used the JMP software (version 15.2; SAS Institute, Cary, NC, USA).

## 3. Results

A total of 191 patients with ICH were admitted to our stroke center during the study period, and 141 patients met our inclusion criteria. Patient characteristics are summarized in Table 1. ICH occurred in 18 patients (12.8%) aged <50 years, in 56 patients (39.7%) aged <65 years, and in 107 patients (75.6%) aged <80 years. 

T-CHO and TG were negatively correlated with the age at onset of ICH and positively correlated with the concentrations of most FAs. However, their correlation with FA wt% was not constant (Table 2). Age was negatively correlated with the concentrations of the majority of FAs and FA wt% values, except for EPA and DHA (Table 3). 

After excluding FA concentrations with multicollinearity and those without a correlation with age, we performed multiple linear regression analysis using the LaA, LiA, DGLA, AA, and EPA concentrations as explanatory variables. The analysis revealed that DGLA and EPA were independent variables when the age at onset of ICH was an objective variable, and a D–W ratio of 1.82 indicated the appropriateness of the linear regression (Table 4). We also performed multiple linear regression analysis using the LaA wt%, OlA wt%, DGLA wt%, AA wt%, and EPA wt% values as explanatory variables and found that DGLA wt% and EPA wt% were independent variables when the age at onset of ICH was an objective variable, and a D–W ratio of 1.88 indicated the appropriateness of the linear regression (Table 4).

After excluding the EPA concentration and DGLA wt% because of multicollinearity (Table 3), we performed multiple linear regression analysis using the DGLA concentrations and the EPA wt%, which demonstrated that the DGLA concentration and EPA wt% were independent variables when age at onset of ICH was an objective variable, and the D–W ratio of 1.85 indicated the appropriateness of linear regression (Table 4). Multiple logistic regression analyses of the DGLA concentration and EPA wt% revealed that the DGLA concentration (*p* = 0.005) and EPA wt% (*p* = 0.002) were significant variables when the objective variable was the age of onset < 65 years (AUC: 0.761). The EPA wt% was a significant variable when the objective variable was the age of onset < 50 years (AUC: 0.829). The DGLA concentration was a significant variable when the objective variable was the age of onset < 80 years (AUC: 0.793). ROC curves showed estimated threshold values of ≥86.7 µmol/L for the DGLA concentration for onset at age < 80 years, ≥108.6 µmol/L for the DGLA concentration and ≤1.7% for the EPA wt% for onset at age < 65 years, and ≤1.3% for the EPA wt% for onset at age < 50 years (Table 5).

## 4. Discussion

Our results show that in patients with ICH, the DGLA concentration and EPA wt% are significant independent variables for the age at onset of ICH and that increased DGLA concentrations and decreased EPA proportion are significantly associated with young-onset ICH. Decreased EPA proportion is a significant variable for ICH onset at age < 50 years; decreased EPA proportion and increased DGLA concentration are significant variables for ICH onset at age < 65 years, and increased DGLA is a significant variable for ICH onset at age < 80 years. 

Patients with young onset of ICH had higher levels of T-CHO, TG, and FAs compared to those with old-onset of ICH. This relationship is likely due to the amount of dietary T-CHO and TGs, including FAs. Patients with an older age at the onset of ICH had lower levels of T-CHO, TGs, and FAs. Therefore, lower T-CHO and TGs might be associated with ICH [4].

Except for EPA and DHA, FA concentrations were positively correlated with the age at onset of ICH; the levels of LiA and DGLA (n-6 PUFAs) were strongly correlated with the levels of PA and StA (SFAs) as well as of OlA (an n-9 MUFA). Strong correlations were observed between various SFA concentrations. OlA concentration was strongly correlated with the levels of SFAs, LiA, DGLA, AA (n-6 PUFAs), and AlA (n-3 PUFA). EPA concentration was strongly correlated with that of DHA (Table 3). These strong mutual correlations were likely due to the ingestion of certain foods, including large amounts of various FAs, as their recent dietary quantities would influence their serum concentrations at admission.

Moreover, the wt% values of FAs, which reflected the dietary FA composition, might be influenced not by the quantity of ingested FAs but by their quality. The wt% values of seven FAs (LaA, MyA, OlA, DGLA, AA, EPA, and DHA) were correlated with the age at onset of ICH, and EPA wt% was strongly correlated with the DHA wt%. Correlations between EPA concentration and wt% with DHA concentration and wt% were likely to be associated with intake of fish or seafood. The FA wt% values displayed different trends compared to the FA concentrations.

In a study with healthy Canadians in their 20s, the median concentrations of DGLA and EPA were 68.2 and 32.4 μmol/L, respectively [15]; these are lower than the concentrations detected in our study. In particular, the median EPA concentration in our study was 176.8 μmol/L, which is much higher than that in healthy young people. In a study of patients with CAD in the United States with an average age of 47 years, the wt% of DGLA and EPA were 0.4% and 0.9%, respectively [12]. The proportions of DGLA and EPA were lower in CAD patients in the US than in the subjects in our study. The average age of patients in our study was 67 years, and 60.3% of our patients were aged 65 years or older. EPA wt% of 2.0% in patients with ICH was higher than that of 0.9% in the US CAD patients. The EPA wt% was almost the same as that of an average of 1.9% in healthy Japanese people [11].

It has been suggested that n-3 PUFAs can reduce the incidence of CAD and stroke, as well as the mortality associated with cardiovascular disease [16,17,18,19,20]. In contrast, SFAs might increase the risk of these conditions [21]. In our patients, the concentration and proportion of SFAs were not independent variables for age at the onset of ICH; however, DGLA concentration and EPA wt% were independent variables, and elevated DGLA concentration and decreased EPA wt% values were associated with young-onset ICH. EPA wt% of 1.3% or less was the threshold for onset at age < 50 years in our study; however, our patients were East Asian, mostly Japanese, and other races might have different threshold values. 

Dietary intake of TGs, which are found in meat, fish, vegetables, and their oils, influences serum FA concentrations and proportions. We did not investigate the dietary intake of FAs by the subjects, before the onset of ICH. Hence, it is essential to assess the types and quantities of dietary TG sources consumed during the days before ICH onset. Assessment of dietary intake and the development of dietary regimens that decrease DGLA concentration and increase the EPA proportion could prevent ICH or delay its onset. 

### Limitations

Our study has several limitations. A small number of patients were included, and the study was retrospective and cross-sectional. As our patients were East Asians, there might be racial differences in the association of DGLA and EPA with ICH, and in the threshold values of DGLA and EPA for the ICH age of onset. In order to determine the effects of DGLA or EPA concentrations and proportions on the delay of ICH onset or ICH prevention, a prospective randomized control study using a food frequency questionnaire and serum FA levels is required.

## Figures and Tables

**Table 1:** Patient characteristics.

	*n* = 141
Age (mean ± SD) (min, max) years	67.4 ± 14.4 (min: 22, max: 95)
Male sex (*n*, %)	87 (61.7%)
Height (mean ± SD) cm	162.0 ± 8.8
Body Weight (MD, IQR) kg	61 (54–70)
BMI (MD, IQR) kg/m^2^	23.6 (21.0–25.7)
Statin users (*n*, %)	24 (17%)
Glucose and lipids	
Glucose (MD, IQR) mmol/L	6.88 (5.90–8.55)
Hemoglobin A1c (MD, IQR) % (NGSP)	5.8 (5.5–6.3)
Total cholesterol (MD, IQR) mmol/L	5.25 (4.69–5.97)
Low-density lipoprotein cholesterol (MD, IQR) mmol/L	2.97 (2.29–3.57)
High-density lipoprotein cholesterol (MD, IQR) mmol/L	1.56 (1.23 2013 1.88)
Triglycerides (MD, IQR) mmol/L	1.18 (0.75–1.92)
Saturated fatty acids	
Lauric acid (LaA) (MD, IQR) μmol/L	5.49 (2.99–10.73)
Myristic acid (MyA) (MD, IQR) μmol/L	80.59 (56.72–120.67)
Palmitic acid (PA) (MD, IQR) μmol/L	2574 (2169–3215)
Stearic acid (StA) (MD, IQR) μmol/L	677.6 (566.9–827.6)
LaA (MD, IQR) wt%	0 (0–0.1)
MyA (MD, IQR) wt%	0.6 (0.5–0.9)
PA (MD, IQR) wt%	23.4 (22.4–24.4)
StA (MD, IQR) wt%	6.8 (6.2–7.4)
n-9 MUFA	
Oleic acid (OlA) (MD, IQR) μmol/L	2140 (1662–2724)
OlA (MD, IQR) wt%	21.3 (19.3–23.6)
n-6 PUFAs	
Linoleic acid (LiA) (MD, IQR) μmol/L	2.696 (2.248–3.134)
Dihomo-gamma-linolenic acid (DGLA) (MD, IQR) μmol/L	101.7 (74.5–137.1)
Arachidonic acid (AA) (MD, IQR) μmol/L	527.8 (447.4–652.1)
LiA (MD, IQR) wt%	26.5 (24.0–28.5)
DGLA (MD, IQR) wt%	1 (0.8–1.3)
AA (MD, IQR) wt%	5.6 (4.9–6.5)
n-3 PUFAs	
Alpha-linolenic acid (AlA) (MD, IQR) μmol/L	69.3 (52.8–105.7)
Eicosapentaenoic acid (EPA) (MD, IQR) μmol/L	176.8 (106.6–255.7)
Docosahexaenoic acid (DHA) (MD, IQR) μmol/L	361.2 (272.2–467.2)
AlA (MD, IQR) wt%	0.7 (0.6–0.9)
EPA (MD, IQR) wt%	2.0 (1.2–2.7)
DHA (MD, IQR) wt%	4.4 (3.3–5.3)
EPA/AA ratio (MD, IQR)	0.32 (0.19–0.47)
n-6/n-3 ratio (MD, IQR)	4.67 (3.60–6.54)

A1c, the A1c fraction of hemoglobin type A; BMI, body mass index; IQR, interquartile range; min, minimum; max, maximum; MD, median; NGSP, National Glycohemoglobin Standardization Program; n-3 PUFA, *n*-3 polyunsaturated fatty acid; n-6 PUFA, n-6 polyunsaturated fatty acid; n-9 MUFA, *n*-9 monounsaturated fatty acid; SD, standard deviation; wt%, weight percentage of total fatty acids.

**Table 2:** Spearman correlation coefficients. Correlation coefficients between T-CHO, TG, age at ICH onset, and FA concentrations and proportions.

***r_s_***	**Age**	**LaA**	**MyA**	**PA**	**StA**	**OlA**	**LiA**	**DGLA**	**AA**	**AlA**	**EPA**	**DHA**
T-CHO	−0.17	0.13	0.26	0.45	0.46	0.32	0.51	0.41	0.46	0.34	0.20	0.38
TG	−0.31	0.44	**0.62**	**0.70**	0.55	**0.71**	0.48	0.59	0.49	0.57	0.09	0.27
***r_s_***		**LaA%**	**MyA%**	**PA%**	**StA%**	**OlA%**	**LiA%**	**DGLA%**	**AA%**	**AlA%**	**EPA%**	**DHA%**
T-CHO		−0.03	−0.01	−0.16	0.05	−0.09	0.11	0.17	−0.06	0.14	0.01	0.06
TG		0.19	0.39	0.15	−0.15	0.54	−0.34	0.19	−0.29	0.35	−0.19	−0.22

AA, arachidonic acid; AlA, alpha-linolenic acid; DHA, docosahexaenoic acid; DGLA, dihomo-gamma-linolenic acid; EPA, eicosapentaenoic acid; ICH, intracerebral hemorrhage; LaA, lauric acid; LiA, linoleic acid; MyA, myristic acid; OlA, oleic acid; PA, palmitic acid; *r_s_*, Spearman correlation coefficient; StA, stearic acid; T-CHO, total cholesterol; TG, triglyceride; Bold values denote strong correlation.

**Table 3:** Spearman correlation coefficients. Correlation coefficients between the age of ICH onset and FA concentrations.

*r_s_*	Age	LaA	MyA	PA	StA	OlA	LiA	DGLA	AA	AlA	EPA	DHA
Age		−0.38	−0.27	−0.36	−0.40	−0.36	−0.38	−0.48	−0.27	−0.20	0.27	0.11
LaA	−0.38		**0.81**	0.59	**0.64**	0.55	0.48	0.56	0.34	0.49	−0.06	0.02
MyA	−0.27	**0.81**		**0.81**	**0.75**	**0.74**	0.58	**0.65**	0.49	**0.69**	0.13	0.28
PA	−0.36	0.59	**0.81**		**0.82**	**0.93**	**0.75**	**0.68**	0.68	**0.72**	0.19	0.44
StA	−0.40	**0.64**	**0.75**	**0.82**		**0.78**	**0.77**	**0.67**	**0.63**	**0.67**	0.19	0.40
OlA	−0.36	0.55	**0.74**	**0.93**	**0.78**		**0.74**	**0.66**	**0.61**	**0.72**	−0.05	0.33
LiA	−0.38	0.48	0.58	**0.75**	**0.77**	**0.74**		0.55	0.50	**0.76**	−0.03	0.26
DGLA	−0.48	0.56	**0.65**	**0.68**	**0.67**	**0.66**	0.55		0.50	0.41	−0.20	0.02
AA	−0.27	0.34	0.49	**0.68**	**0.63**	**0.61**	0.50	0.50		0.36	0.19	0.38
AlA	−0.20	0.49	**0.69**	**0.72**	**0.67**	**0.72**	**0.76**	0.41	0.36		0.23	0.41
EPA	0.27	−0.06	0.13	0.19	0.19	−0.05	−0.03	−0.20	0.19	0.23		**0.78**
DHA	0.11	0.02	0.28	0.44	0.40	0.33	0.26	0.02	0.38	0.41	**0.78**	
**Correlation Coefficients between the Age at Onset of ICH and FA Percentages**												
*r_s_*	Age	LaA%	MyA%	PA%	StA%	OlA%	LiA%	DGLA%	AA%	AlA%	EPA%	DHA%
Age		−0.25	−0.14	−0.04	−0.08	−0.25	0.09	−0.27	0.10	−0.02	0.42	0.40
LaA%	−0.25		0.65	0.12	0.34	0.13	−0.11	0.16	−0.17	0.13	−0.15	−0.30
MyA%	−0.14	0.65		0.27	0.22	0.22	−0.34	0.25	−0.22	−0.29	−0.10	−0.23
PA%	−0.04	0.12	0.27		−0.23	0.23	−0.60	0.06	0.01	−0.27	0.01	−0.01
StA%	−0.08	0.34	0.22	−0.23		−0.31	0.08	0.29	0.06	−0.10	0.04	0.00
OlA%	−0.25	0.13	0.22	0.23	−0.31		−0.37	0.07	−0.30	0.18	−0.48	−0.46
LiA%	0.09	−0.11	−0.34	−0.60	0.08	−0.37		0.00	−0.10	0.15	−0.26	−0.24
DGLA%	−0.27	0.16	0.25	0.06	0.29	0.07	0.00		0.12	−0.16	−0.42	−0.39
AA%	0.10	−0.17	−0.22	0.01	0.06	−0.30	−0.10	−0.12		−0.52	0.15	0.18
AlA%	−0.02	0.13	−0.29	−0.27	−0.10	0.18	0.15	−0.16	−0.52		−0.01	−0.04
EPA%	0.42	−0.15	−0.10	0.01	0.04	−0.48	−0.26	−0.42	0.15	−0.01		**0.83**
DHA%	0.40	−0.30	−0.23	−0.01	0.00	−0.46	−0.24	−0.39	0.18	−0.04	**0.83**	
**Correlation coefficients between the four significant variables**												
*r_s_*	DGLA	EPA	DGLA%	EPA%								
DGLA		−0.20	**0.73**	−0.20								
EPA	−0.20		−0.42	**0.91**								
DGLA%	**0.73**	−0.42		−0.42								
EPA%	−0.20	**0.91**	−0.42									

AA, arachidonic acid; AlA, alpha-linolenic acid; DHA, docosahexaenoic acid; DGLA, dihomo-gamma-linolenic acid; EPA, eicosapentaenoic acid; ICH, intracerebral hemorrhage; LaA, lauric acid; LiA, linoleic acid; MyA, myristic acid; OlA, oleic acid; PA, palmitic acid; StA, stearic acid, Bold values denote strong correlation.

**Table 4:** Multiple linear regression analysis of the age at onset of ICH.

**Significant FA Concentrations as Explanatory Variables**	***t*-Value**	***p***	**Adjusted R^2^**	**Durbin–Watson Ratio**
			0.254	1.82
Lauric acid (LaA)	−0.30	ns		
Linoleic acid (LiA)	−1.84	ns		
Dihomo-gamma-linolenic acid (DGLA)	−3.43	<0.001		
Arachidonic acid (AA)	−0.48	ns		
Eicosapentaenoic acid (EPA)	2.02	<0.05		
**Significant FA Percentages as Explanatory Variables**	***t*-Value**	***p***	**Adjusted R^2^**	**Durbin–Watson Ratio**
			0.190	1.88
LaA wt%	−1.40	ns		
Oleic acid wt%	−1.38	ns		
DGLA wt%	−2.07	<0.05		
AA wt%	0.80	ns		
EPA wt%	2.12	<0.05		
**DGLA and EPA wt% as Explanatory Variables**	***t*-Value**	***p***	**Adjusted R^2^**	**Durbin–Watson Ratio**
			0.238	1.85
DGLA	−4.40	<0.0001		
EPA%	2.30	<0.05		

FA, fatty acid; ICH: intracerebral hemorrhage; ns, not significant; wt%, weight percentage.

**Table 5:** Threshold values for the age at onset of ICH using receiver operating curves from logistic regression analysis.

	*N*	Sens (%)	Spec (%)	PPV (%)	Odds Ratio	*p*	AUC	AICc	BIC
Age <50 years as an OV									
EPA wt% (≤1.3 vs. >1.3) %	141	83.3	74.0	31.9	0.28 (0.12–0.56)	<0.0001	0.812	95	101
Age <65 years as an OV									
DGLA (≥108.6 vs. <108.6) µmol/L	141	66.1	74.1	62.7	1.02 (1.01–1.03)	<0.0001	0.718	173	179
EPA wt% (≤1.7 vs. >1.7) %	141	67.9	71.8	61.3	0.46 (0.30–0.66)	<0.0001	0.736	171	177
Age < 80 years as an OV									
DGLA (≥86.7 vs. <86.7) µmol/L	141	77.6	76.5	91.2	1.03 (1.02–1.05)	<0.0001	0.793	130	136

AICc, corrected Akaike information criterion; AUC, area under the curve; BIC, Bayesian information criterion; DGLA, dihomo-gamma-linolenic acid; EPA, eicosapentaenoic acid; ICH, intracerebral hemorrhage; OV, objective variable; PPV, positive predictive value; Sens, sensitivity; Spec, specificity; wt%, weight percentage.
